# Epidemiology of food allergy in the community

**DOI:** 10.1186/2045-7022-1-S1-S47

**Published:** 2011-08-12

**Authors:** Kirsi Jarvinen

**Affiliations:** 1 Mount Sinai School of Medicine, Pediatric Allergy and Immunology, New York, USA

## 

Food allergy is the most common cause of anaphylaxis outside the hospital setting. Peanut, tree nuts, milk, egg, and shellfish are the most commonly implicated foods. Most cases of anaphylaxis are reported to occur in the home, but one-fifth of the reactions occur at school or work and a similar proportion in restaurants and other food establishments. Although the majority of reactions in schools occur in subjects with known food allergies, nearly 25% of peanut/tree nut-induced allergic reactions were reported before the diagnosis. The majority of reactions occur in the classroom (many due to craft projects), followed by health office, playground, and cafeteria. Numerous studies have identified deficiencies in establishing and implementing written emergency action plans (EAP) and general or individualized plans for prevention in schools, and inadequacies in recognizing and treating anaphylaxis. Although there are no studies directly comparing the effect of banning peanut schools on the incidence of reactions, such practice from preschool to lower elementary school is not uncommon. Reactions to nuts occurring in food establishments most commonly include Asian food restaurants, ice cream parlors and bakeries/dough nut shops. Allergen is commonly found in dessert foods or hidden in sauce, dressing etc. Cross-contamination during preparation and serving is another important source of error, in addition to reactions due to ingestion of food not intended for the subject, buffet items, or casual contact. Importantly, the establishment is often not notified of the allergy. Lack of timely treatment with epinephrine is a universal risk factor and adolescents/young adults are the peak age group for a fatal food-induced anaphylaxis. Furthermore, up to one-fifth of food-induced anaphylactic reactions may need more than 1 dose of epinephrine. Therefore, there is an urgent need for education and access to epinephrine in food-induced reactions occurring in the communal setting.

